# Prevalence of salivary epstein-barr virus in potentially malignant oral disorders and oral squamous cell carcinoma

**DOI:** 10.4317/medoral.20785

**Published:** 2016-01-31

**Authors:** Leticia Bagan, María-Dolores Ocete-Monchon, Manuel Leopoldo-Rodado, Judith Murillo-Cortes, Jose-M. Díaz-Fernández, Rafael Medina-Gonzalez, Concepción Gimeno-Cardona, Jose-V. Bagan

**Affiliations:** 1 Associate Professor of Oral Medicine, European University of Valencia, Spain; 2 Consultant Microbiology Service. University General Hospital, Valencia; 3 Consultant in Oral and Maxillofacial Surgery. Service of Stomatology and Maxillofacial Surgery, Valencia; 4 Head Section in Oral and Maxillofacial Surgery. Service of Stomatology and Maxillofacial Surgery, Valencia; 5 Professor and Head Service of Microbiology. Valencia University and University General Hospital Valencia; 6 Professor and Head Service of Stomatology and Maxillofacial Surgery. Valencia University and University General Hospital Valencia, Spain

## Abstract

**Background:**

To analyze the presence of salivary Epstein-Barr virus (EBV) DNA in oral squamous cell carcinoma and potentially malignant oral disorders.

**Material and Methods:**

Three groups were studied: Group 1 (12 oral squamous cell carcinomas (OSCC)), Group 2 (12 potentially malignant oral disorders (PMD)) and Group 3 (47 healthy controls). EBV DNA salivary analysis was performed by PCR.

**Results:**

The highest percentage of positive salivary EBV DNA corresponded to the OSCC group (58.3%), followed by the PMD group (41.7%) and the controls (40.4%). The differences between groups were not statistically significant, however (*p*>0.05).

**Conclusions:**

Salivary EBV DNA was more prevalent in OSCC than in PMD or the controls.

**Key words:**EBV DNA, saliva, oral squamous cell carcinoma, oral leukoplakia.

## Introduction

Epstein-Barr virus (EBV) is a very well known oncogenic human herpes virus that has been implicated in several malignant tumors affecting epithelial cells and B lymphocytes (1).

The prevalence of EBV in the general population is very high, and there are nearly 200,000 new cases of infection in the world every year. In the first two decades of life EBV infects 90% of all individuals. According to Ueda *et al*., EBV is a reversible latent infection in B cells (2).

It is believed that EBV initially penetrates and multiplies within epithelial cells, followed by release into saliva, affecting B lymphocytes and spreading throughout the rest of the body. However, most affected individuals are asymptomatic, despite detection of the virus in different body secretions and blood (3).

Saliva plays a significant role in the capacity of EBV to become transmitted to other people. The virus is infective when present in saliva, both in asymptomatic and symptomatic carriers. EBV is released into saliva from the epithelial cells, and it is in this fluid where maximum infectious capacity is observed (1). On the other hand, EBV is found not only in B cells but also in nasopharyngeal carcinomas (4).

Salivary EBV has been analyzed in several oral diseases, particularly in patients with periodontal problems (5-9). In 2007 we already addressed the presence of EBV in potentially malignant disorders (PMD) and in oral squamous cell carcinoma (OSCC), though involving a smaller number of cases (10). However, there have been recent controversial findings in the literature regarding the association between EBV and OSCC. The present study analyzes the frequency of EBV DNA positivity in OSCC and PMD comparing with controls.

## Material and Methods

Three groups were studied: Group 1 (12 OSCC), Group 2 (12 PMD) and Group 3 (47 healthy controls).

There were no clinical differences among the three groups regarding age or gender (*p*>0.05).

Oral squamous cell carcinoma was diagnosed from an incisional biopsy. In the leukoplakia group (Group 2) we also obtained a biopsy to establish the diagnosis following the criteria of Carrard *et al*. (11).

The study was approved by the Ethics Committee of the University of Valencia (Spain), and informed consent was obtained from each patient.

Whole non-stimulated saliva was obtained in all cases according to the criteria of Navacek *et al*. (12). Saliva samples were immediately stored and frozen at -80º until EBV DNA analysis.

EBV DNA salivary analysis was performed by PCR following the methodology described elsewhere (3). Saliva samples were obtained and DNA was extracted as reported (2). EBV DNA levels were determined by qualitative real-time PCR (qPCR) targeting the EBV Gen LMP1 region.

The present case-control study evaluated the presence and percentage of positive findings regarding EBV DNA and analyzed the association of the virus to the different groups using the χ2 test. Statistical significance was considered for *p* < 0.05.

## Results

The highest percentage of positive salivary EBV DNA corresponded to the OSCC group, followed by the leukoplakia (PMD) group and the controls ( Table 1). The differences between groups were not statistically significant, however (*p*>0.05) ( Table 2).

**Table 1:**
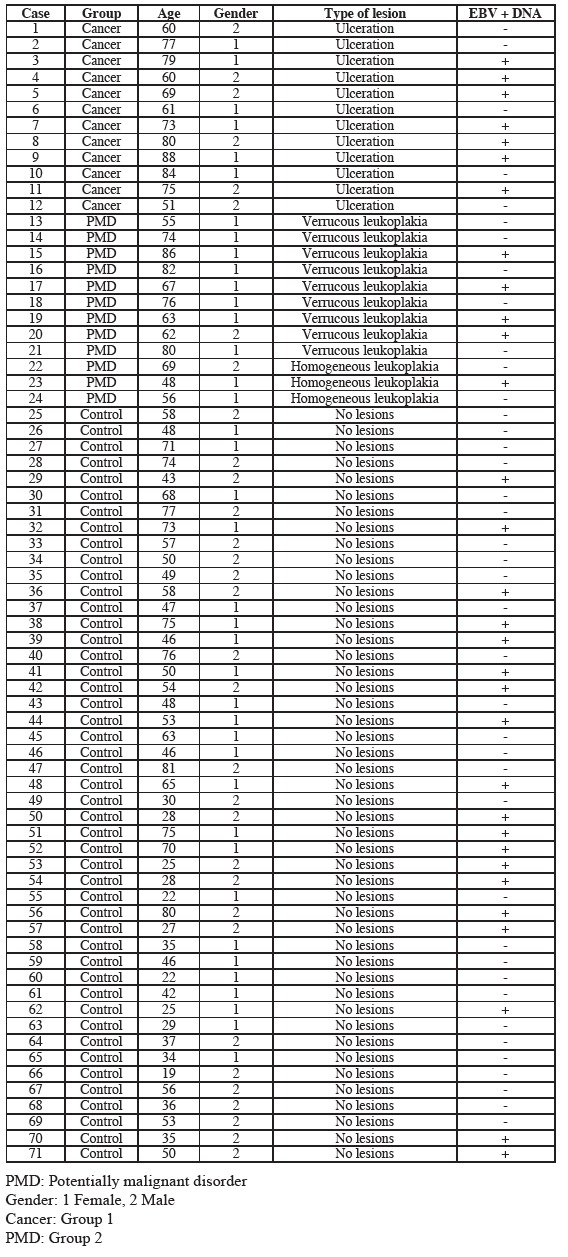
Epstein-Barr virus DNA findings among the three groups.

**Table 2:**
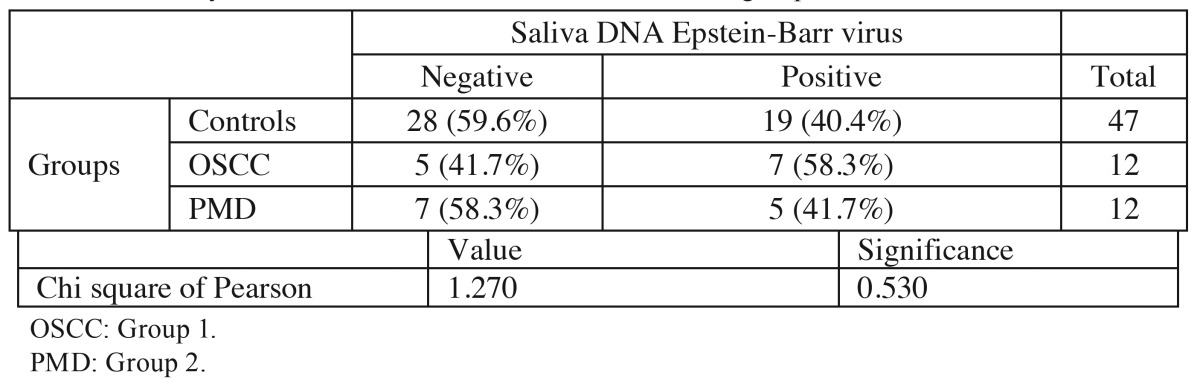
Summary of DNA EBV detection in saliva of the three groups.

Four of the 9 cases of proliferative verrucous leukoplakia (44.4%) presented positive salivary EBV DNA. In those cases with only homogeneous leukoplastic areas, the positivity rate was lower (33.3%).

## Discussion

Epstein-Barr virus is very common in normal individuals of the general population. According to Ueda *et al*. (2), its prevalence may reach 90% in saliva. The virus can penetrate and multiply within the epithelial cells, followed by release into saliva.

The salivary EBV DNA detection rate and consequently shedding of the virus in healthy persons ranges from 22-90% (3). Despite the variability among authors, the detection rate is usually high in healthy controls. In this respect we found 40.4% of our controls to be positive for DNA EBV.

Epstein-Barr virus DNA in saliva has been analyzed in several diseases such as connective tissue disorders (13), adverse drug reactions with eosinophilia and systemic symptoms (DRESS) (14), periimplantitis (15), HIV infection (3,16), periodontal disease (6) and in transplant patients (17).

In cancer patients, EBV in saliva has been described as a useful tool in nasopharyngeal carcinomas. In advances disease stages the EBV DNA levels are higher than in early stages (18).

Epstein-Barr virus has also been studied in OSCC patients, though the results are controversial. According to some investigators, EBV is associated to OSCC and this association seems to be enhanced by betel quid chewing, thus suggesting that EBV may be an important etiological risk factor for OSCC (19). Furthermore, Jiang *et al*. (20) described a high prevalence of human papillomavirus (HPV)/EBV infection and coinfection in non-cancerous base of tongue (BOT) lesions and tonsil malignancies, possibly reflecting their origins in lymphoid-rich tissue (20).

In contrast, other authors have found no significant OSCC risk in subjects with EBV infection (21,22). Likewise, the data published in 2014 by Saravani *et al*. (23) neither supported the hypothesis that EBV and HHV-6 are directly involved in OSCC nor ruled out the possibility that these viruses might play an indirect carcinogenic role in this area.

Considering the above discrepancies, we tried to analyze the presence of EBV DNA in the saliva of patients with potentially malignant disorders and oral squamous cell carcinoma. No significant differences were observed among OSCC, PMD and the controls (*p*>0.05). However, EBV DNA positivity was greater in the OSCC group than in the PMD group or controls ( Table 2). Studies with a larger number of cases are required to determine whether such higher percentage EBV DNA positivity in OSCC is also found in other larger populations.
